# Association of COVID-19 Infection with Sociodemographic, Anthropometric and Lifestyle Factors: A Cross-Sectional Study in an Older Adults’ Population Aged over 65 Years Old

**DOI:** 10.3390/diseases11040165

**Published:** 2023-11-09

**Authors:** Eleni Pavlidou, Sousana K. Papadopoulou, Georgios Antasouras, Theofanis Vorvolakos, Olga Alexatou, Gerasimos Tsourouflis, Exakousti-Petroula Angelakou, Aspasia Serdari, Maria G. Grammatikopoulou, Evmorfia Psara, Konstantinos Vadikolias, Antonios Dakanalis, Nikolaos Lefantzis, Constantinos Giaginis

**Affiliations:** 1 Department of Food Science and Nutrition, School of Environment, University of Aegean, 81400 Myrina, Greece; elen.p.pavl@gmail.com (E.P.); g.antasouras@gmail.com (G.A.); rd.olga.alexatou@gmail.com (O.A.); xeniaggelakou@hotmail.com (E.-P.A.); fnsd21013@fns.aegean.gr (E.P.); cgiaginis@aegean.gr (C.G.); 2 Department of Nutritional Sciences and Dietetics, School of Health Sciences, International Hellenic University, 57400 Thessaloniki, Greece; 3 Department of Geriatric Psychiatry, University Hospital of Alexandroupolis, Democritus University of Thrace, 68100 Alexandoupoli, Greece; tvorvola@med.duth.gr; 4 Second Department of Propedeutic Surgery, Medical School, National and Kapodistrian University of Athens, 11527 Athens, Greece; gtsourouflis@med.uoa.gr; 5 Department of Psychiatry and Child Psychiatry, University Hospital of Alexandroupolis, Democritus University of Thrace, 68100 Alexandroupoli, Greece; aserdari@yahoo.com; 6 Unit of Immunonutrition and Clinical Nutrition, Department of Rheumatology and Clinical Immunology, School of Health Sciences, Faculty of Medicine, University of Thessaly, 41110 Larissa, Greece; mgrammat@uth.gr; 7 Department of Neurology, School of Medicine, Democritus University of Thrace, 68100 Alexandroupoli, Greece; kvadikol@med.duth.gr; 8 Department of Mental Health and Addiction, Fondazione IRCCS San Gerardo dei Tintori, 20900 Monza, Italy; antonios.dakanalis@unimib.it; 9 Department of Medicine and Surgery, University of Milan Bicocca, Via Cadore 38, 20900 Monza, Italy; 10 Department of Oral and Maxillofacial Surgery, Medical School, Attikon Hospital, National and Kapodistrian University of Athens, Chaidari, 12462 Athens, Greece; dlefa@yahoo.gr

**Keywords:** COVID-19 infection, sociodemographic factors, anthropometric factors, depression, anxiety, stress, cognitive impairment, sleep quality, health-related quality of life, Mediterranean diet

## Abstract

Background: The COVID-19 pandemic has led to unfavorable disruptions to daily living routines by exerting deleterious effects on several aspects of human mental and physical health and quality of life worldwide. The purpose of the current survey is to explore the potential association of COVID-19 infection with multiple sociodemographic, anthropometric, and lifestyle factors of community-dwelling older adults. Methods: This is a cross-sectional survey including 5197 older adults aged over 65 years old from 10 geographically diverse regions of Greece. Relevant questionnaires were used to record study the population sociodemographic factor, while anthropometric parameters were also measured. Validated questionnaires were also applied to assess several lifestyle factors such as depression, anxiety, stress, cognitive status, sleep quality, health-related quality of life, physical activity levels, and Mediterranean diet (MD) adherence. Results: In multivariate regression analysis, COVID-19 infection was significantly, independently related with urban residence (*p* = 0.0107), regular smoking (*p* = 0.0218), overweight status and obesity (*p* = 0.0036), as well as abdominal obesity (*p* = 0.0008), higher risk of depression (*p* = 0.0027), anxiety (*p* = 0.0045), stress (*p* = 0.0038), inadequate sleep quality (*p* = 0.0108), lower physical activity levels (*p* = 0.0012), reduced MD compliance (*p* = 0.0009), and poor health-related quality of life (*p* = 0.0002). In univariate analysis, older adults’ age (*p* = 0.0001), male gender (*p* = 0.0015), living alone (*p* = 0.0023), lower educational and economic level (*p* = 0.0175 and *p* = 0.0294, respectively), and cognition decline (*p* = 0.0032) were also related with the presence of COVID-19 infection; however, these associations were considerably attenuated at a non-significant level by adjustment for several confounders in multivariate analysis. Conclusions: This is one of the few available studies supporting evidence that COVID-19 infection may be associated with diverse sociodemographic, anthropometric, and lifestyle factors in an older adults’ population in Greece. This study highlights the strong demand to provide psychological and nutritional counselling and support to older adults diagnosed with COVID-19 infection in order to ameliorate disease symptoms and severity, emphasizing the adaptation of healthy dietary and lifestyle habits as preventing and supplementary therapeutic factors against COVID-19.

## 1. Introduction

Global society has detrimentally been influenced by the coronavirus disease of 2019 (COVID-19) that increasingly developed to a pandemic on 11 March 2020 [1,2]. The emergence of the COVID-19 pandemic has resulted in unfavorable disruptions of daily living routines by exerting deleterious effects on human mental and physical health and overall quality of life worldwide, which were mostly ascribed to the distress, ambiguity, and loneliness people experienced [3,4]. Several substantial research studies have shown that in the initial wave of the COVID-19 pandemic, mental health (i.e., depressive symptoms, anxiety, general distress, etc.), physical activity, sleep quality, and daily quality of life had significantly been worsened than prior to the pandemic, while many persons experienced negative emotional effects due to the fear of contagion and the unexpected death of relatives or friends due to COVID-19 infection or its short- or long-term complications [5,6]. The detrimental medical complications, the gradually elevated incidence of morbidity, and the significantly increasing international spread of COVID-19 have resulted in important public health problems, motivating multiple governments as well as political measures and strategies across all the world [7]. To slow down the gradually increasing spread of disease, almost all countries were forced to establish rigorous health guidelines and social disconnection strategies [8,9].

Several risk factors have been recognized to affect COVID-19 infection, such as socioeconomic, anthropometric, and lifestyle factors [10]. Several studies have demonstrated that health outcomes like the probability of developing a disease and then dying are highly associated with the socioeconomical state [11]. Studying viral pulmonary disorders and the 1918, 1919, and 2009 influenza pandemics had previously highlighted a higher likelihood of contracting the disorder and dying among socioeconomically disadvantaged people [12,13]. Notably, loneliness, distress, and feelings of social disconnection were highly increased in all age groups due to the COVID-19 pandemic, which were mainly ascribed to the political energies to reduce the COVID-19 infection spread by establishing isolation actions and stay-at-home commands [14]. Older adults already felt alone or perceived themselves to be in social isolation before the COVID-19 pandemic and were at a higher probability of morbidity and mortality, such as cases of cardiovascular diseases, mental health, and cognitive decline [15,16,17,18,19]. Amongst older peoples, COVID-19 confinement further increased feelings of aloneness, sadness, and perceived social isolation. The above negative effects were found to be more common in women, those living alone, and those with low socioeconomic status, as well as older adults during COVID-19 quarantine [15,16,17,18,19].

It has been well described that pneumonia due to a coronavirus has been clearly identified as severe acute respiratory syndrome coronavirus 2 (SARS-CoV-2). Nevertheless, it has been recognized more commonly as COVID-19, which was firstly noticed in Wuhan, China, and it has been considered as extremely infectious [20]. COVID-19 basic clinical symptomatology involves fever, fatigue or myalgia, dry cough, breath shortness, and breathing difficulties [21,22]. COVID-19 infection appears to differ from mild to severe disorders which could lead to acute respiratory distress syndrome and multi-organ decline and death [21,22,23]. Alarmingly enough, COVID-19 seems to negatively influence other organs such as the brain, and recent studies on neurological symptomatology due to COVID-19 infection are currently emerging [24,25]. Thus, it is reasonable that worldwide, the deaths due to COVID-19 mostly concern older adults usually presenting more than one comorbidity [26].

Several sociodemographic factors have been associated with depression and/or anxiety and stress, such as living alone, lower educational level, living mainly in urban areas, and female gender; however, the above data remain inconsistent, while the available reports on age as a risk factor are also contradictory [27,28]. Τhere are several indications that COVID-19 infection may increase the levels of post-traumatic stress symptoms, resulting in an elevated probability of depression, anxiety, psychological distress, and poor sleep quality as well as enhanced likelihood of psychiatric symptoms and/or quality of life; however, the available research data still remain inconclusive regarding mainly the direct impacts of COVID-19 infection in several aspects of mental health [29,30,31,32]. Concerning the lack of direct effects of COVID-19 on general mental health, it appears to elevate depression, anxiety, and stress symptomatology in combination with adverse effects on general mental health [29,30,31,32]. The COVID-19 pandemic has also adversely affected physical activity by almost a half of the people worldwide, which has been associated with a considerable increase in body weight, leading to a higher incidence of overweight condition and obesity and/or abdominal obesity [33].

It should be emphasized that there has been an emerging issue that older adults have been at a difficulty throughout pandemic as it is less possible to get efficient information concerning COVID-19, and they have exhibited inadequate admission to the internet and social media, also presenting cognitive disturbances [34,35,36]. Worryingly enough, older adults who have been detached from their healthcare personnel may exhibit lower and inadequate access to novel information about COVID-19 and thus have less of an understanding of the requirement to take suitable medication in the case of COVID-19 infection [37,38]. In support of this view, in a recent cross-sectional study, the incidence of social loneliness amongst older people in the community reached 44.3%. The overall social loneliness in community-dwelling older people was considerably related with a higher risk of malnourishment risk, a worse quality of life, and with higher probability of developing depressive symptoms [39]. In a recent cohort study conducted from 2019 to 2021, the frequency of undergone isolation was enhanced amongst older home-dwellers from 26% to 30% [40]. In fact, throughout the 2 years of the pandemic, the feelings of aloneness and mood disturbances were elevated even after adjustment by diverse confounding factors [40]. Notably, social isolation and reduced physical activity were identified as potential risk agents for negative health outcomes in the older adults’ population [41,42]. The above risk factors have been related with poor quality of life, decreased muscle mass, worse cognition functionality, multimorbidity, and disability, higher prevalence of falls and depression, a higher risk of hospitalization, and mortality [41,42].

As far as Greece is concerned, there is little research concerning the effect of the COVID-19 pandemic and confinement on mental and physical health and in daily life of their citizens. Importantly, a substantial study has shown that 21.3% of Greek citizens were > 65 years old, and therefore, our country presents the second-eldest population in Europe, right below Italy (22%) [43]. In Greece, confirmed COVID-19–infected people present a mean age of 49 years old, and more than half of the infected patients were males [43]. In addition, the available statistics have shown that 190 COVID-19–infected individuals were dead from disease (38 women and 92 men) with a mean age of 74 years [33,43]. Moreover, 90% of the above deceased infected people had several other comorbidities and/or an age of 70 years or above [43,44].

However, most of the currently available research studies in our country as well as worldwide concern children, adolescents, young adults, and university students and middle-aged adults, whereas research data regarding older adults still remain scarce and are mainly restricted to hospitalized or institutionalized older persons. Moreover, the most available research studies did not concern the potential effects of healthy dietary patterns such as Mediterranean diet (MD) against COVID-19 infection, its symptom severity, and its complications in older adults. In Greece, such studies have not been available yet. In this aspect, the current survey constitutes one of the few studies in Greece that aims to explore the effect of COVID-19 infection in diverse sociodemographic, anthropometric, and lifestyle factors of community-dwelling older Greek people from ten geographically various areas of Greece and to assess whether a healthy dietary pattern such as MD may be associated with lower COVID-19 infection symptoms’ severity and complications.

## 2. Methods

### 2.1. Study Population

**Figure 1:**
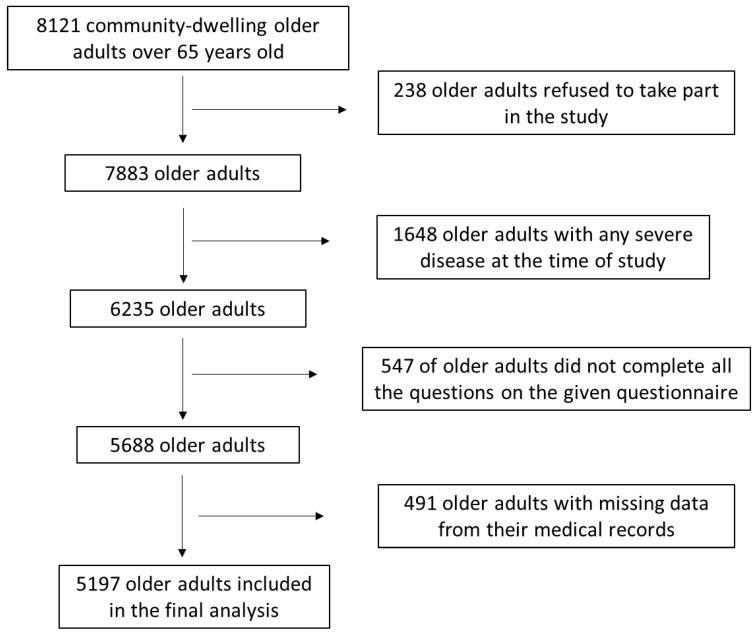
Flow chart diagram of study enrolment.

Initially, 8121 community-dwelling elder Caucasian adults aged ≥ 65 years old were arbitrarily enrolled, living in 10 geographically different regions of Greece, including urban and rural areas, namely Athens, Thessaloniki, Alexandroupoli, Larissa, Ioannina, Patra, Kalamata, Crete, and North and South Aegean. Enrollment to the survey was between June 2020 and October 2022. The enrolled older adults were mostly found at the time of their visits at healthcare centers for routine check-up and in public units associated with entertaining events for older adults. A detailed description of the survey assignment as a flow chart diagram is presented in Figure 1. By applying diverse appropriate exclusion and inclusion criteria, 5197 older adults were contained in the present study, leading to a reply rate of 64.0%.

This survey was taken with approval by the Ethical Agency of the University of the Aegean (ethical approved protocol: 8/21.1.2020, date of approval: 22 January 2020) and was in agreement with the World Health Organization (52nd WMA General Assembly, Edinburgh, Scotland, UK, 2000). All assigned older adults’ data were strictly private and anonymous. All enrolled older adults were given detailed information concerning the aim of the survey and signed a consent form accepting the potential publication of the study results. Sample size estimation was conducted by the use of PS: Power and Sample Size calculator program 3.1 (Developer: William D. Dupont and Walton D. Plummer, Jr., Informer Technologies, Inc., Los Angeles, CA, USA, https://ps-power-and-sample-size-calculation.software.informer.com/3.1/ (accessed on 17 May 2020). The randomization was performed utilizing an order of arbitrary binary numbers (i.e., 001110110 in which 0 presented assignment and 1 not assignment to the survey). Simple randomization is a study methodology that assigns participants to treatment groups by chance rather than by the choice of someone involved with the program. The calculation of the power of our sample size indicated a power equal to 88.6%.

### 2.2. Study Design

This is a cross-sectional survey that has assessed several sociodemographic parameters such as age, gender, employment status (employed vs. unemployed), type of residence (urban vs. rural regions), living status (living with others vs. living alone), educational and economic level, and smoking habits (smokers vs. never smokers) of the enrolled older adults infected or not infected by COVID-19. All sociodemographic data were self-reported through one-to-one interviews between the assigned older adults and qualified personnel to reduce memory biases. The education status was categorized into three classes as: (a) primary education, (b) secondary education, and (c) university studies. Economic level according to the yearly income was categorized as: as low for yearly income ≤10,000 EUR, medium for yearly income >10,000 EUR and ≤20,000 EUR, and high for yearly income >20,000 EUR.

Body Mass Index (BMI) was determined by measuring body weight and height at the time of study. Participants’ body weight was determined utilizing a Seca scale [Seca, Hanover, MD, USA], without shoes, to the nearest 100 g, and height was determined utilizing a portable stadiometer (GIMA Stadiometer 27335, Gima, Ponte San Giovanni, Italy) with no shoes on, to the nearest 0.1 cm. The WHO criteria were used to categorize the enrolled older individuals into three groups: (a) normal weight, (b) overweight, and (c) obese [45]. The waist circumference was determined at the midpoint between the inferior margin of the last palpable ribs and the upper point of the iliac crest, while the hip circumference was determined near the broadest portion of the buttocks, with the tape parallel to the floor [46]. The Waist Hip Ratio (WHR) was estimated through dividing waist dimension by hip dimension. WHR has been found to be superior to BMI [46]. It has been recognized as a greater indicator of abdominal obesity, which is considered as a better anthropometric measure for more efficiently estimating the probability of various cardiometabolic disorders [46].

Eight qualified and authorized questionnaires were created for evaluating depressive behavior, health-related quality of life, cognitive status, anxiety, stress, sleep quality, physical activity levels, and MD adherence of the enrolled older adults. Particularly, depressive behavior was evaluated utilizing the effective Geriatric Depression Scale (GDS) questionnaire that includes 30 questions [47]. For every question, there were 2 potential responses, “yes” or “no”, assessed as 0 or 1, respectively. Total GDS scoring was estimated by the sum of 30 component scores. An overall GDS score ≥15 is indicative of depressive behavior [47].

Health-Related Quality of Life (HRQOL) was evaluated by the use of the Short Form Healthy Survey (SF-36) questionnaire that contains 36 questions, evaluating the health state via 8 subscales [48]. The initial 4 subscales determine the physical HRQOL: physical functionality, physical role limitations, body pain, overall health assessment. The 2nd 4 subscales evaluate mental HRQOL: role limitations because of emotional disturbances, vital energy, mental health, social functioning. For all the above subscales, a scoring varying between 0 (worst) and 100 (best) is estimated. All but one item are allocated to one of the 8 health fields screening diverse aspects of physical and mental health: physical functionality (PF, 10 items), physical role functionality (RP, 4 items), body pain (BP, 2 items), general health insights (GH, 5 items), vitality, (VT, 4 items), social role functioning (SF, 2 items), emotional role functionality (RE, 3 items), and mental health (MH, 5 items) [48]. HRQOL was further grouped into two summary dimensions: the physical component summary (PCS) and the mental component summary (MCS) [48].

The well-recognized and effective Mini Mental State Examination (MMSE) questionnaire was used to determine the cognitive state of the enrolled older adults [49]. The MMSE is efficient as a testing instrument for identifying cognition decline in older, community-dwelling, hospitalized, and institutionalized adults. It is a 30-question tool which evaluates 5 domains of cognition functionality: orientation, registration, attention and calculation, recall, and language. The upper limit score is 30. A score of 23 or lower is indicative of cognition decline. In fact, a score between 20 and 23 is indicative of mild cognitive impairment, while a score less than 20 is indicative of moderate or severe cognitive impairment [49,50].

The 6-item short-form State–Trait Anxiety Inventory (STAI-6) was applied to assess the anxiety of participants [51]. This is a consistent and valid instrument with acceptable reliability and validity as well as precision concerning variations in state anxiety [51]. It is also likely to increase response rates and decrease the proportion of answer mistakes and unanswered items, therefore enhancing the validity and generalizability of any results [51]. The above scale includes 6 questions and 4 potential responses (1 = not at all, 2 = somewhat, 3 = moderately, and 4 = very much) which represent usual signs of anxiety experienced by an individual. The scores on the 3 positively worded questions were reverse-coded, and a greater scoring corresponded to an elevated level of individual anxiety [51,52].

The Perceived Stress Scale (PSS) was applied to determine stressful life incidents and conditions which tend to stimulate or worsen disorder symptomatology [53,54]. This questionnaire contains 10 questions and is the greatest broadly used psychological tool to determine the perception of stress. It is an evaluating tool of the level to which conditions in one’s life are classified as stressful. Questions were constructed to evaluate in what way the lives of respondents are unpredictable, uncontrollable, and overloaded. This scale further contains various direct inquiries concerning the present degree of felt stress. It is considered as a widely used tool for assisting us to recognize how different conditions influence our feelings and our perceived stress. The items of the above scale ask about your feelings and thoughts throughout the previous month. Particularly, the older adults are questioned to reveal how often his/her felt or thought a specific way. Although certain questions seem similar, there are differences among them, and the participants have to respond to each one as an independent question. Individual scores on the PSS are calculated with values between 0 and 40, with greater scores reflecting elevated perceived stress. Scores with values between 0–13 are classified as low stress. Scores with values between 14–26 are categorized as moderate stress. Scores between 27–40 are classified as high perceived stress [53,54].

We further assessed sleep quality by utilizing the well-recognized Pittsburgh Sleep Quality Index (PSQI) that includes 19 questions, is scored on a 4-point scale (0–3), and is classified into seven components (sleep quality, sleep latency, sleep period, habitual sleep effectiveness, sleep disruption, taking sleeping medicines, and daytime disfunction) [55]. The responses’ scores in every component were added and transformed to component scores with values from 0 (better) to 3 (worse) according to the related guidelines [55]. Total PSQI scoring was determined as the summation of the seven component scores with values between 0 and 21, where greater scoring shows inferior sleep quality. An overall PSQI scoring of <5 is representative of adequate sleep quality [55].

We also assessed physical activity levels, utilizing the widely used International Physical Activity Questionnaire (IPAQ). In the above questionnaire, the participants report the extent of exercise performed in a normal week. The above self-reported questionnaire, which is widely utilized, determines the total physical activity during the previous week, and classifies physical activity levels as low, moderate, or high [56]. IPAQ tools have comprehensively been evaluated and demonstrated good reliability and appropriate authority properties, being equivalent to other self-reported PAQs [56].

MD compliance was determined by the use of the certified MedDietScore [57,58]. The above questionnaire assesses the foodstuffs intake occurrence of 11 particular food classes according to the Med Diet Score index. Each item contains 6 potential replies, scoring from 0 to 5, that depend on the level of adherence for every food’s component. The sum of the 11 replies leads to a scoring with values from 0 to 55. A higher scoring is indicative of elevated MD adherence [57,58]. Regarding cereals, potatoes, fruits, vegetables, dairies, and olive oil, the levels of 6 possible replies corresponds to daily consumption. As far as legumes, fish, red meat, and poultry concerns, the rates of 6 probable replies correspond to weekly intake [57,58]. The 11th item determines wine consumption at a daily incidence with moderate consumption (≤1 and ≤2 drinks/day for women and men, respectively; one drink = 100 mL = 12 g ethanol), being classified as the highest score [57,58]. The enrolled individuals were classified into quartiles according to their MedDietScore. A MedDietScore below 23 shows very low MD adherence, and a MedDietScore between 23–26 indicates low MD adherence. A MedDietScore between 27–30 shows moderate MD adherence, while a MedDietScore 31 and above indicates high MD adherence.

All questionnaires were fulfilled with the assistance of qualified medical personnel (e.g., medical and nursing personnel) as well as nutritionists and dietitians by face-to-face interviews to reduce recall biases. The qualified personnel have provided detailed information to the enrolled older peoples for all the questionnaires to increase the reliability and accuracy of their responses.

### 2.3. Statistical Analysis

Student’s *t*-test was applied for continuous variables that were normally distributed. Kolmogorov–Smirnov test was used to determine whether the examined variables followed the normal distribution. Chi-square test was utilized for categorical variables. The quantitative variables, following normal distribution, are stated as mean value ± standard deviation (SD). Absolute or relative frequencies were used for the qualitative variables. The quantitative variables that did not follow normal distribution are stated as a median value (interquartile range, IQR range). Multivariate binary logistic regression was utilized to evaluate if COVID-19 infection could be related at an independent level with the recorded sociodemographic, anthropometric, and lifestyle factors after adjustment for confounders. As confounding factors, we included all the available variables in multivariate analysis as all of them could have a confounding effect. Multivariate regression findings are presented as relative risk (RR) and 95% confidence intervals (CI). Differences were judged as significant at *p* < 0.05 and 95% confidence interval. Statistica 10.0 software, Europe (Informer Technologies, Inc., Hamburg, Germany) was utilized to perform the statistical analysis of the study data.

## 3. Results

### 3.1. Association of COVID-19 Infection with Sociodemographic and Anthropometric Parameters of the Enrolled Older Adults

Among the assigned older individuals, 42.5% of them were infected by COVID-19. Older peoples with COVID-19 infection had a significantly higher mean age compared to non-infected older adults (Table 1, *p* = 0.0001). Male participants were significantly more frequently infected by COVID-19 than female participants (Table 1, *p* = 0.0015). Older adults living in urban regions were significantly more often infected by COVID-19 compared to older adults living in rural regions (Table 1, *p* = 0.0006). Participants who lived alone exhibited a considerably elevated prevalence of being infected by COVID-19 compared to participants who lived with others (Table 1, *p* = 0.0023). A lower education level was considerably related with a higher prevalence of COVID-19 infection (Table 1, *p* = 0.0175). A lower economic level was also significantly associated with an elevated frequency of COVID-19 infection (Table 1, *p* = 0.0294). Smoker older adults were significantly more frequently infected by COVID-19 than never-smoker older adults (Table 1, *p* = 0.0016). Based on BMI classification, overweight and obese older adults were significantly more frequently infected by COVID-19 than normal weight older adults (Table 1, *p* ˂ 0.0001). In addition, based on WHR classification, abdominal obesity was significantly more often observed in infected older adults compared to older adults without abdominal obesity (Table 1, *p* ˂ 0.0001).

### 3.2. Association of COVID-19 Infection with Lifestyle Factors of the Enrolled Older Adults

A substantial elevated incidence of depression was recorded in COVID-19 infected older adults compared to non-infected ones (Table 1, *p* = 0.0021). A better health-related quality of life (HRQOL) status was noted in healthy participants than those infected by COVID-19 (Table 1, *p* ˂ 0.0001). Higher scores of PCS and MCS were more frequently observed in healthy participants than those infected by COVID-19 (Table 1, *p* = 0.0003 and *p* = 0.0005, respectively). COVID-19–infected older adults were considerably associated with a higher incidence of cognitive impairment than healthy older adults (Table 1, *p* = 0.0032). Non-infected participants also exhibited a significantly better sleep quality than those infected by COVID-19 (Table 1, *p* ˂ 0.0001). Anxiety and stress were significantly more often observed in COVID-19–infected older adults than healthy older adults (Table 1, *p* = 0.0003 and *p* ˂ 0.0001, respectively). Healthy older adults also significantly exhibited higher physical activity levels compared to COVID-19–infected older adults (Table 1, *p* ˂ 0.0001). Participants with higher levels of MD adherence were significantly associated with a lower prevalence of COVID-19 infection compared to participants adopting lower levels of MD (Table 1, *p* ˂ 0.0001).

### 3.3. Multivariate Binary Logistic Regression Analysis Examining Whether COVID-19 Infection May Exert an Independent Effect in Sociodemographic, Anthropometric, and Lifestyle Factors

In multivariate binary logistic regression analysis, COVID-19 infection was independently related with type of residence, smoking habits, BMI and WHR status, depression, HRQOL, sleep quality, anxiety, stress, physical activity levels, and MD adherence (Table 2, *p* ˂ 0.05). The associations of older adults’ age, gender, living status, educational and economic level, and cognitive status in unadjusted analysis, were considerably attenuated in multivariate analysis, being insignificant (Table 2, *p* > 0.05).

Older adults living in urban regions had a 38% elevated prevalence of being infected by COVID-19 compared to participants who lived in rural regions (Table 2, *p* = 0.0107). Regular smoker participants exhibited a 72% elevated incidence of being infected by COVID-19 than never-smoker older adults (Table 2, *p* = 0.0218). Older adults affected by overweight condition and obesity showed more than two-fold higher frequency of presenting COVID-19 infection compared to normal weight older adults (Table 2, *p* = 0.0036). Older adults presenting abdominal obesity also had a more than two-fold elevated incidence of having a diagnosis of COVID-19 infection compared to those without abdominal obesity (Table 2, *p* = 0.0008).

Participants infected by COVID-19 showed a 59% elevated prevalence of presenting depressive symptomatology than non-infected older adults (Table 2, *p* = 0.0027). Older adults infected by COVID-19 had more than a two-fold probability of exhibiting a harmful health-related quality of life (HRQOL) than non-infected older adults (Table 2, *p* = 0.0002). Participants infected by COVID-19 exhibited a 34% elevated likelihood of presenting poor sleep quality compared to non-infected older adults (Table 2, *p* = 0.0108). Participants infected by COVID-19 showed a 79% enhanced probability of presenting anxiety compared to non-infected older adults (Table 2, *p* = 0.0045). Accordingly, COVID-19–infected older adults showed about a two-fold higher risk of presenting stress compared to non-infected older adults (Table 2, *p* = 0.0038). Participants infected by COVID-19 showed a 73% elevated probability of adopting lower physical activity levels than non-infected older adults (Table 2, *p* = 0.0012). Moreover, COVID-19–infected older adults exhibited a more than two-fold enhanced likelihood of adopting lower MD adherence compared to non-infected older adults (Table 2, *p* = 0.0012). Almost identical results were obtained if, instead of HRQOL, PCS and MCS scores were included in the multivariate model.

## 4. Discussion

The present cross-sectional study has finally enrolled 5197 older adults during a period of about two years by using relevant inclusion and exclusion criteria. During the period of study population recruitment, 42.5% of the enrolled older adults were found positive for COVID infection with the majority of them to present mild disease symptoms (modest fever, fatigue, mild myalgia, occasional dry cough, sporadic breath shortness, and some of them moderate breathing difficulties). COVID-19 infection was considerably related at an independent level with urban residence, regular smoking, overweight condition and obesity, as well as abdominal obesity, higher risk of depressive symptoms, anxiety, stress, inadequate sleep quality, lower physical activity levels, reduced MD compliance, and poor health-related quality of life. Older adults’ age, male gender, living alone, lower educational and economic level, and cognitive impairment were also significantly related with the presence of COVID-19 infection in unadjusted analysis; however, these associations were considerably attenuated in multivariate analysis, being non-significant.

Among the results of the current survey, those concerning the harmful effects of COVID-19 infection in several aspects of mental health should be considered of major importance since they can significantly exert deleterious effects on daily quality of life of older citizens. COVID-19 infection may negatively affect different organs of the human body like the breathing system, gastrointestinal tract, kidneys, and central nervous system (CNS) [59,60,61]. It is currently well known that COVID-19 virus can enter the CNS via diverse paths, leading to symptoms such as dizziness, headache, seizures, loss of consciousness, depressive behavior, posttraumatic stress disorder, memory impairment, insomnia, sleep disorder, and anxiety [59,60,61]. Depression constitutes the most frequent disease amongst all neurological/neurodegenerative symptoms following COVID-19 infection; however, the potential mechanisms of COVID-19–related depression remain still unclear. In COVID-19 infected older adults, most depression symptoms can be evidently observed during the period of disease as well as after a partial recovery period [62,63]. Both anxiety- and depression-related COVID-19 infection may worsen the disease prognosis, and also exert a considerable harmful impact on the immune system [64]. In addition, several substantial pieces of evidence have demonstrated that the incidence of depression in individuals with COVID-19 infection is about 45%, for anxiety 47%, and for sleeping disturbances 34% [65]. The above findings are in accordance with our results.

Among the most frequent neuropsychiatric complications of COVID-19 infection is anxiety. Several biological and psychosocial risk factors increasing the prevalence of anxiety have been identified in individuals with COVID-19 infection [66]. Biological risk factors contain stress, resilience, genetics, gender, age, immune system, direct infection of the CNS with SARS-CoV-2, comorbid psychiatric and general medical illnesses, acute respiratory distress syndrome, and intensive care unit stay [67,68]. Anosmia and hypogeusia have been identified as COVID-19–specific anxiety risk factors and knowledge of the anxiety risk factors is crucial to perform appropriate interventions, as anxiety could be a complication that may extremely impair the COVID-19 course [66,67]. An interesting cross-sectional online survey has determined the impact of social, demographical, and psychological factors in predicting anxiety, stress, and concern of COVID-19 infection in older peoples at the first and the second waves of the COVID-19 pandemic in Slovakia [69]. Six hundred and seven older adults (Sample 1) and 156 older adults (Sample 2) were enrolled in this survey during the first and second waves of the COVID-19 pandemic, respectively. The participants in both groups have shown moderate levels of anxiety, stress, and concern of COVID-19 infection [68]. Moreover, high rates of variability in anxiety and stress were recorded, which were ascribed to potential powerlessness, intolerance of uncertainty, optimism, and coping self-efficacy [68]. The above findings are in accordance with several relevant clinical surveys performed on cohorts of older peoples in diverse countries throughout the COVID-19 pandemic period [70,71,72,73].

In our study, we found a significantly elevated probability of poor sleep quality in older adults infected by COVID-19 than non-infected older adults. In this aspect, several studies are in accordance with our findings. Specifically, COVID-19 induced loneliness has resulted in several health disturbances such as diminished sleep, while the loneliness–sleep relationship was very high amongst those with more COVID-19–associated fears or amongst those with reduced resilience [74]. It has been shown that in older peoples who feel strong fears concerning COVID-19, the impacts of loneliness on their sleep may be very harmful [74]. Another study has documented that sleep latency, sleep disturbances, and daytime dysfunction have significantly been harmful in individuals with COVID-19 infection [75]. Also, it has been reported that several infected individuals have suffered from major insomnia or sleep disruptions and impaired cognition function [76]. In a recent survey on COVID-19 survivors, individuals with a previous serious disease have shown an incidence of 29% for sleep disturbances [77].

Moreover, COVID-19 lockdowns and lifestyle change due to the pandemic have increased obesity rates. This is another serious concern which has been ascribed to the deleterious alterations in daily life patterns, which were ascribed to enforced social isolation and intense worry of infection as well as physical inactivity due to the closure of gyms, gymnastic entertainment, and fitness centers. Obesity increases the risk of several diseases because of the excess adipose tissue increases in the human body. The prevalence of obesity has constantly elevated because of the increasing rates of physical inactivity and behavioral distances due to COVID-19 epidemic [78,79]. Notably, a recent study has documented that 32.9% of its participants showed low physical activity because of COVID-19 confinement, while 42.1% of them showed extensive body weight gain [80]. Characteristically, the incidence of adult obesity elevated from 30% in 2014 to 34.6% in 2018 and physical activity reduced from 58.3% in 2014 to 47.6% in 2018. The above observations were additionally worsened by COVID-19 confinements [80]. The World Health Organization (WHO) [81] has acknowledged COVID-19 and body weigh increase as an extreme pandemic and emphasized the significance to establish preventive measures [79]. Social loneliness measures enhanced harmful nutritional habits and reduced exercise incidence, leading to general unhealthy behaviors that may result in adiposity increase and obesity, while people affected by obesity appear to be more susceptible to be infected by COVID-19 [82]. Notably, during UK lockdown, older people who engaged in risky health behaviors were at higher risks of weight gain and obesity both in the short run and long term [83]. During the COVID-19 pandemic, U.S. adult obesity rates were higher and worsened the pre-existing epidemic of adult obesity in the U.S. [84]. Characteristically, the WHO just released in May 2022 a report on the state of the obesity pandemic in Europe, stating that 60% of citizens in the area of Europe are either overweight or obese, and highlighting the implications of the obesity pandemic, especially as it interacts with the COVID-19 pandemic to create a twin pandemic, to increase morbidity and mortality [85].

Notably, a substantial review including 44 surveys from 18 countries worldwide has supported that obesity can increase the probability of major COVID-19 complications, intensive care units’ entry, intubation, and mortality [86,87]. Patients with overweight or obesity appear to be more susceptible to SARS-CoV-2 infection, while more severe disease may require entry to intensive care unit and intubation, also being associated with elevated mortality incidences [86,87]. Accordingly, a systematic review and meta-analysis has also demonstrated that as body weight elevated, the probabilities of hospitalization, intensive care units’ entry, and requirement for invasive mechanical ventilation elevated, mainly in COVID-19–infected individuals affected by obesity [88]. The above evidence is in accordance with our findings which have shown that overweight and obese older adults as well as those presenting abdominal obesity exhibited a considerably greater risk to be infected by COVID-19.

It should be noted that the immune system’s function and the possibility of interactions with foods have received special attention during the effort to control COVID-19 infection, providing a better functional response and a more effective protection against COVID-19 infection [89,90]. Immune system response has highly been regulated by inflammatory cascades and oxidative stress and can determine the intrinsic defense process to block pathogens from invading the body such as virus or bacteria [91,92]. It has been reported that adequate nutrients’ intake can provide some amount of protection against the virus and may assist in managing the infection in the event of illness [93,94]. In addition, nutrition exhibits an influence on human microbiome and may be related with the probability of acquiring a virus, and developing an infection [95,96]. A balanced diet such as MD and an optimal nourishing state are crucial agents, which can affect immunological potency among individuals of all ages; however, only a small number of surveys so far have investigated the impact of nutritional habits in association with the probability of being infected by COVID-19. In accordance with our findings, Sharma et al. have provided evidence that the implementation of a well-adjusted MD, including increased intake of cereals, elevated olive oil consumption, moderate alcohol consumption, and greater consumption of fruits and nuts, could be a crucial method to reduce the likelihood of COVID-19 infection [97]. In a case-control study, individuals infected by COVID-19 have shown a reduced mean MedDiet score than non-infected individuals [98]. Perez-Araluce et al., also shown that MD appears more effective compared to each of its constituents in preventing COVID-19 infection and symptomatology [99]. In the ‘Seguimiento Universidad de Navarra’ cohort, it was also found that a better MD adherence could be related with a lesser probability of COVID-19 infection [100].

Accordingly, an observational study conducted on 900 healthy adults has documented considerably lower levels of MedDietscore, with a lower intake of plant-based foodstuffs in individuals infected by COVID-19 [101]. It has also been assumed that MD could be a possible effective strategy to prevent adverse conditions related with COVID-19 infection and severity, including diabetes mellitus, cardiovascular diseases, and obesity [102,103]. Several other studies have also provided substantial evidence that a higher compliance to MD, plant-based diets and pescetarian diets has been related with a reduced probability and intensity of COVID-19 symptomatology among healthy adults [104,105]. Moreover, it should be emphasized that aging is a normal procedure which has directly been associated with oxidation conditions (e.g., oxidation of nucleotide DNA bases, suppression of DNA repairing processes, release of reactive oxygen species, destructions of telomeres, epigenetic alterations, etc.) and inflammation triggering (e.g., suppression of pro-inflammatory agents, decline in malondialdehyde and superoxide dismutase levels, suppression of cyclooxygenases enzymes, decline in thrombocytes, and white blood cells amount, etc.) [106,107]. In this context, the favorable healthy impacts of MD have mainly been attributed to its foodstuffs’ components like fruits, legumes, vegetables, olive oil, herbs, spices, and fibers that include high amounts of various bioactive ingredients, presenting several antioxidant and anti-inflammatory activities [108,109]. The numerous antioxidant and anti-inflammatory MD components may enhance immune system, leading to prevention of virus spreading, precluding the disorder development to advanced progression, and additionally suppressing the highly inflammation, promoting both preventive and treatment care against COVID-19 [89].

Oxidative stress and inflammation have been considered to exert crucial impacts in the pathogenesis of COVID-19 infection [110,111]. Notably, the generation of abnormal levels of oxidants under a COVID-19-induced cytokine storm may lead to the irreversible oxidation of a wide range of macromolecules and subsequent damage to cells, tissues, and organs, resulting in endothelial damage, which may increase the risk of complications in COVID-19 and post-COVID-19 or long-COVID-19 cases [110]. COVID-19 severity related with cytokine storm phenomenon can also lead to the activation of inflammatory cascades, which is a common disease feature [111]. The activation of the nuclear factor kappa light-chain-enhancer of activated B cells (NF-κB) pathway is central to pro-inflammatory signaling driven by SARS-CoV-2 infection and may underlie increased susceptibility to COVID-19 progression. Specifically, NF-κB is the priming signal for nucleotide-binding oligomerization domain (NOD)-like receptor (NLR) family pyrin domain containing 3 (NLRP3) inflammasome activation [112]. In this aspect, the antioxidant and anti-inflammatory ingredients of MD may slow down COVID-19 infection progression, reducing its symptoms severity, and minimizing post-COVID-19 complication.

## 5. Conclusions

This is one of the few available studies so far supporting evidence that COVID-19 infection may be associated with diverse sociodemographic, anthropometric, and lifestyle factors in an older adults’ population. This study emphasizes the strong demand to provide psychological and nutritional counselling and support to older adults infected by COVID-19 to ameliorate disorder symptoms and severity. Public policies and strategies are strongly suggested to advise healthy older peoples about the potential risk factors that may increase the likelihood of COVID-19 infection, highlighting the adaptation of healthy dietary and lifestyle habits such as MD adherence as preventing and supplementary therapeutic factors against COVID-19 infection.

## Figures and Tables

**Table 1:** Association of COVID-19 infection with sociodemographic, anthropometric, and lifestyle factors of the enrolled study population.

Parameters (n = 5197)	COVID-19 Infection		*p*-Value
	No 2987 (57.5%)	Yes 2210 (42.5%)	
**Age** (mean ± SD years)	71.8 ± 7.3	74.9 ± 7.8	*p* = 0.0001
**Gender (n, %)**			*p* = 0.0015
Male	1373 (46.0%)	1124 (50.9%)	
Female	1614 (54.0%)	1086 (49.1%)	
**Employment (n, %)**			*p* = 0.0833
Employed	502 (16.8%)	332 (15.0%)	
Unemployed	2485 (83.2%)	1878 (85%)	
**Type of residence (n, %)**			*p* = 0.0006
Urban	1735 (58.1%)	1483 (67.1%)	
Rural	1252 (41.9%)	727 (32.9%)	
**Living status (n, %)**			*p* = 0.0023
Living with others	2419 (81.0%)	1483 (67.1%)	
Living alone	568 (19.0%)	727 (32.9%)	
**Educational level (n, %)**			*p* = 0.0175
Primary education	993 (33.2%)	846 (38.3%)	
Secondary education	635 (21.3%)	630 (28.5%)	
University studies	1359 (45.5%)	734 (33.2%)	
**Family economic status (n, %)**			*p* = 0.0294
Low	1730 (57.9%)	1329 (60.1%)	
Medium	760 (25.4%)	623 (28.2%)	
High	497 (16.6%)	258 (11.7%)	
**Smoking habits (n, %)**			*p* = 0.0016
Smokers	1806 (60.5%)	1507 (68.2%)	
Never smokers	1181 (39.5%)	703 (31.8%)	
**BMI status (n, %)**			*p* ˂ 0.0001
Normal Weigh	2354 (78.8%)	1471 (66.6%)	
Overweight	452 (15.1%)	496 (22.4%)	
Obese	181 (6.1%)	243 (11.0%)	
**WHR (n, %)**			
Low	2144 (71.8%)	1158 (52.4%)	*p* ˂ 0.0001
Medium	602 (20.1%)	698 (31.6%)	
High	241(8.1%)	354 (16.0%)	
**Depression (n, %)**			*p* = 0.0021
Yes	873 (29.2%)	783 (35.4%)	
No	2114 (70.8%)	1427 (64.6%)	
**HRQOL score (mean ± SD)**	53.7 ± 11.3	49.9 ± 11.1	*p* ˂ 0.0001
**PCS score (mean ± SD)**	52.1 ± 11.1	49.5 ± 11.2	*p* = 0.0003
**MCS score (mean ± SD)**	49.5 ± 11.6	47.2 ± 11.8	*p* = 0.0005
**Cognitive status (n, %)**			*p* = 0.0032
No cognitive impairment	2108 (70.6%)	1354 (61.3%)	
Mild cognitive impairment	510 (17.1%)	465 (21.0%)	
Moderate/severe cognitive impairment	369 (12.3%)	391 (17.7%)	
**Sleep quality (n, %)**			*p* ˂ 0.0001
Adequate	2035 (68.1%)	1310 (59.3%)	
Inadequate	952 (31.9%)	900 (40.7%)	
**Anxiety (n, %)**			*p* = 0.0003
No	2072 (69.4%)	1403 (63.5%)	
Yes	915 (30.6%)	807 (35.5%)	
**Stress (n, %)**			*p* ˂ 0.0001
Low	2016 (67.5%)	1282 (58.0%)	
Moderate	757 (25.3%)	653 (29.5%)	
High	214 (7.2%)	275 (12.4%)	
**IPAQ status (n, %)**			*p* ˂ 0.0001
Low	1493 (50.0%)	1346 (60.9%)	
Medium	835 (27.9%)	633 (28.1%)	
High	659 (22.1%)	243 (11.0%)	
**MedDietScore (n, %)**			*p* ˂ 0.0001
Very low	450 (15.1%)	854 (38.6%)	
Low	500 (16.7%)	784 (35.5%)	
Moderate	970 (32.5%)	329 (14.9%)	
High	1067 (35.7%)	243 (11.0%)	

**Table 2:** Multivariate analysis assessing whether COVID-19 infection may independently be affected by sociodemographic, anthropometric, and lifestyle factors of the enrolled study population.

Participants’ Characteristics	COVID-19 Infection (No vs. Yes)	
	OR * (95% CI **)	*p*-Value
**Age** (Below/Over mean value)	1.58 (1.02–2.11)	*p* = 0.1376
**Gender** (Female/Male)	1.13 (0.61–1.88)	*p* = 0.2463
**Employment** (Employed/Unemployed)	1.28 (0.71–1.87)	*p* = 0.2576
**Type of residence** (Rural/Urban)	1.38 (1.06–1.67)	*p* = 0.0107
**Living status** (Living with others/Living alone)	1.25 (0.59–1.91)	*p* = 0.0987
**Educational level** (Primary and secondary education/Universities studies)	1.06 (0.48–1.67)	*p* = 0.3173
**Family economic status** (High/Moderate and low)	1.12 (0.58–1.79)	*p* = 0.4094
**Smoking habits** (No/Yes)	1.72 (1.49–1.96)	*p* = 0.0218
**BMI status** (Normal weight/Overweight + Obesity)	2.08 (1.87–2.39)	*p* = 0.0036
**WHR** (Low/Medium + High)	2.17 (1.98–2.39)	*p* = 0.0008
**Depression** (No/Yes)	1.59 (1.35–1.86)	*p* = 0.0027
**HRQOL** (Over/Below mean value)	2.27 (2.02–2.68)	*p* = 0.0002
**Cognitive impairment** (No/Yes)	1.13 (0.58–1.79)	*p* = 0.1095
**Sleep quality** (Adequate/Inadequate)	1.68 (1.34–2.01)	*p* = 0.0108
**Anxiety** (No/Yes)	1.79 (1.52–2.03)	*p* = 0.0045
**Stress** (Low/Moderate + high)	1.98 (1.73–2.29)	*p* = 0.0038
**IPAQ** (High and moderate/Low)	1.73 (1.48–1.97)	*p* = 0.0012
**Mediterranean diet adherence** (Moderate + High/Very low + Low)	2.22 (1.98–2.45)	*p* = 0.0009

* Odds ratio: OR; ** CI: Confidence Interval.

## Data Availability

The data of the study are available upon request to the corresponding author.
